# Achievement of interventions on HIV infection prevention among migrants in China: A meta-analysis

**DOI:** 10.1080/17290376.2018.1451773

**Published:** 2018-03-22

**Authors:** Rui Zhang, Ling Chen, Ya deng Cui, Ge Li

**Affiliations:** a School of Public Health and Management, Research Center for Medicine and Social Development, Collaborative Innovation Center of Social Risks Governance in Health, Chongqing Medical University, Chongqing, 400016, People’s Republic of China; b The Center of Experimental Teaching Management, Chongqing Medical University, Chongqing, 401331, People’s Republic of China; c School of Public Health and Management, Research Center for Medicine and Social Development, Collaborative Innovation Center of Social Risks Governance in Health, Chongqing Medical University, Chongqing, 400016, People’s Republic of China; d The Center of Experimental Teaching Management, Chongqing Medical University, Chongqing, 401331, People’s Republic of China

**Keywords:** migrants, AIDS/HIV, intervention, meta-analysis, precision intervention

## Abstract

In China, migrants with acquired immunodeficiency syndrome (AIDS) have become a serious problem in the field of AIDS prevention. This study aimed to evaluate the efficacy of interventions for human immunodeficiency virus (HIV) infection prevention for migrants in China and to identify factors associated with intervention efficacy. A computerized literature search of the Chinese National Knowledge Infrastructure, Wan Fang, and PubMed databases was conducted to collect related articles published in China. Only self-control intervention studies or studies containing sections regarding self-control interventions wherein the method of intervention was health education were included. Rev Manager 5.3 software was used to analyze the intervention effects in terms of knowledge, attitude, and behavior indexes. Relative to pre-intervention, the HIV interventions showed statistically significant efficacy in terms of sexual transmission of HIV, condom use for HIV prevention, change in attitude towards HIV/AIDS patients, incidence of commercial sex behavior, and recent condoms use during sex (*P* < .01). Moreover, the baseline rate of migrants, intervention time, peer education, region, and education background were factors influencing the efficacy of the intervention. Significant improvement in terms of knowledge of sexual transmission of HIV and attitudes and behaviors among migrants was observed; however, based on the findings of previous studies, the interventions should be customized for different people from different districts in China. Further research is needed to evaluate subgroups of migrants in China according to their baseline characteristics.

## Introduction

As a malignant disease, acquired immunodeficiency syndrome (AIDS) causes severe harm to human health and hinders economic development. AIDS is characterized by rapid dissemination and high mortality rates. Since AIDS is now globally widespread, it has become a serious public issue and an urgent social problem (Liu & Tang, 2010). Moreover, movement of individuals and populations is an important factor in the spread of the human immunodeficiency virus (HIV) (Soskolne & Shtarkshall, 2002). In many countries, including China, migrants have been identified as a high-risk population of HIV transmission, who may become ‘bridge populations’, transferring HIV from high-risk populations to a low-risk population. In 2016, the number of migrants in China exceeded 245 million (National Health and Family Planning Commission of the People’s Republic of China, 2017), with 48.3% females and 51.7% were males. The average age of migrants was 29.8. In the whole migrants population in China, the generation born in the eighties accounted for 56.5%, and the generation born in the nineties accounted for 18.7%, and this number kept increasing. With rapid urbanization in China and the transfer of surplus rural labor force, population flow has become active unprecedentedly and is currently a prominent social phenomenon. The migrants are defined as populations that have left their domiciles and live in other places (National Health and Family Planning Commission of the People’s Republic of China, 2013). Most migrants are sexually active, and their cultural level is low: their knowledge of sexually transmitted diseases (STDs) and HIV/AIDS and self-awareness are weak. These factors predispose them to several complex sexual and reproductive health risks. In their hometowns, these people are subject to very strict social control. In a strange city, however, this tight external control is weak, making it easier for them to indulge in risky behaviors (Lin, Fang, Lin, Li, & Su, 2006). Combined with China’s urban and rural isolation system, the social status of migrants is very unstable; in addition, immigrant mentality is prominent, which results in them having weak social interactions and poor community inclusion with the urban society (Liu, Ma, Xiong, Li, & Wang, 2002). Thus, migrants tend to display characteristics of marginalized groups and are more likely to practice risky sexual behavior. The prevalence of HIV infection is higher among migrants than among the general population (Fitzgerald et al., 2003). Research conducted by the U.S. Centers for Disease Control and Prevention in 1987 and 1992 reported that the rate of HIV infection among migrants in urban communities was 2.6%–5%, which was 10 times higher than that in ordinary residents (Holmberg, 1996). In China, of the HIV-infected patients detected in urban areas including Shanghai, Shanxi, and Zhejiang from 1995 to 2000, more than two-thirds were migrants (Luo, Mo, & Wu, 2002; National Health and Family Planning Commission of the People’s Republic of China, 2017; Zhu, Wu, & Yu, 2001). Thus, this population is not only at high risk of acquiring HIV infection but also transmitting it. Moreover, because mobility is difficult to manage (Lin, Fang, Li, Xu, & Liu, 2005), migrants with AIDS have become a serious problem in the field of AIDS prevention.

Because of the lack of specific and inexpensive drugs and effective vaccines, health education intervention is considered an effective measure to curb the spread of the AIDS epidemic (Li et al., 2004). Many studies have shown that health education intervention can effectively improve the level of knowledge of AIDS prevention, induce changes in risky behavior, and reduce the spread of HIV infection.

Currently, several studies investigating migrants in the context of HIV prevention have been carried out, but the effects of the interventions have varied greatly. In this study, we used a meta-analysis to evaluate the effect of interventions conducted in previous studies to explore the effects of health education on HIV/AIDS prevention among migrants in China and provide evidence in support of conducting AIDS-related health interventions for migrants.

## Methods

### Search strategy

We performed a systematic literature search using several strategies: (1) electronic database searches, such as PubMed, Wan Fang, and China National Knowledge Infrastructure (CNKI), and keywords such as ‘AIDS’, ‘HIV’, ‘Migrants’, ‘Intervention’, and ‘Health Education’ were used; (2) requested for articles sent to researchers; and (3) reviewed reference sections of articles obtained in the searches. Studies matching the selection criteria and available as of February 10, 2018, were included in the analysis.

### Study selection

Studies were selected if they met the following criteria: (1) they were self-control intervention studies or studies containing sections regarding self-control interventions; (2) they targeted migrants in China; (3) they provided information needed to calculate effect sizes; and (4) the evaluation indicators included HIV/AIDS knowledge, attitude, and behavior change. We excluded studies in which the target study population was sex workers. Duplicate studies, reviews, local reports, conference abstracts, and presentations were excluded. Two co-authors (Rui Z and Ling C) independently extracted relevant studies following the inclusion criteria. In cases of missing data, we contacted the corresponding authors. Disagreements were resolved by consensus in a panel meeting (Rui Z and Ling C). The characteristics of the records included in the meta-analysis are shown in Table 1.

**Table 1.** Summary of the records included in the meta-analysis.

First author	Published year	Region	Sample size (before/after)	Sex (male %)	Age (mean)	Unmarried (%)	Married (%)	Education (junior high school and below, %)	Intervention measures	Outcome indicators
He LP	2010	Hunan (Chenzhou)	210/151	100	38.75/39.24	18.1/16.5	80.0/82.8	8.22/8.63	Panels, posters, condoms, promotional material, knowledge contest	①
Tian Z	2015	Sichuan (Chengdu)	770/738	52.6/53.7	28/27			79.2/78.9	Panels, audiovisual products, promotional material, consults	②③④⑤
Cui YM	2012	Sichuan (Chengdu)	4072/415	55.4/61		12.6/20.6	72.1/75.2	80.1/82.8	Lecture, propaganda, knowledge contest	③
Fei LX	2008	Guangdong (Guangzhou)	1044/1044	15.61	19–39	82.95	11.02	24.39	Lecture, condom use demonstration	①③
Hu LX	2007	Zhejiang (Hangzhou)	298/280	72.48/76.43	30/30	56.71/58.21	41.28/39.64	30.9/36.4	Lecture, panels, consults, condoms, promotional material	①
Wang W	2010	Hebei	1361/1353	98.5/98.9	33.85/33.21	15.7/17.5	82.0/79.7	74.5/76.9	Lecture, panels, propaganda, peer education	③④⑤
Cai Y	2012	Jilin	600/829	93.7/94.8		16.7/19.8	80.6/77.2	89.1/87.5	Peer education, propaganda, consults, condoms, health education	⑤
Zhao CL	2015	Henan (Zhoukou)	865/812	98.6/98.9	35.1/34.8	38.7/38.4	56.5/56.7	77.8/78.1	Lecture, promotional material, panels, health education	②③
Xu H	2006	Hubei (Wuhan)	300/300	100	36.43 ± 10.1		71.5	80.9	Brochures, panels, CD, lecture, knowledge contest	①②
Guo HJ	2009	Jiangsu (Nanjing, Suzhou, Yan-gzhou, Changzhou)	815/728	85.52/83.10	29.6 ± 8.9/30.2 ± 10.0		Married or cohabiting	Junior high school	Self-study, lecture, posters, knowledge contest	①③
Wang YY	2008	Jiangxi (Jiujiang)	973/891						Lecture, propaganda	①
Huang BB	2015	Fujian (Quanzhou, Shishi)	2000/1973			52.4	38.3	85.9	Lecture, consults, propaganda, training, condoms, panels, DVD	②
Xie KP	2011	Xinjiang (Kuitun)	432/369	100	15–49		Married	85	Knowledge contest, propaganda, conversations, peer education, consults	②④⑤
Liang X	2015	Guangxi (Chongzuo)	452/412	64.8	36.7 ± 3.2	68.2	31.8	95.8	Promotional material, lecture	①
Zhong HB	2006	Guangdong (Shantou)	277/242	46.21/46.3	16–58				Brochures, propaganda column, consults, video	①
Li XN	2006	Jiangsu (Wuxi)	767/697	100	26	56.8		68.6	Pamphlets, self-study, lecture, consults comprehensive intervention	①
Peng MY	2015	Guangdong (Guangzhou)	512	0		98.2	1.8	22.1	Propaganda, CD, lecture, message	②③
Hou LY	2011	Shandong (Qiangdao)	861/806	0	22.0 ± 2.8/22.0 ± 2.5	100		58.3/56.1	Pamphlets, condoms, lecture, training, video, hotline	②
Xu L	2011	Shanghai (Nanhui)	657/657	90.4	38.9 ± 10.1	12	84.2	83.57	Pamphlets, posters, panels, DVD, interview	①③
Tian ZY	2010	Jiangsu (Nanjing)	301/235	94.35	36.82 ± 10.02	18.27	73.42	78.1	Lecture, posters	①③
Peng YL	2008	Jiangsu (Nanjing)	1168/1145	46.7/42.6	17–63	15.07/12.14	72.86/71.79	73.0/71.7	Propaganda, lecture, knowledge contest, consults	①③
Zhou QY	2011	Gansu (Tianshui)	345/374	61.16/64.44	25–45	19.42/20.85	80.58/79.15	72.46/73.26	Promotional material, TV, condoms	②
Ma SM	2015	Shanghai (Xuhui)	116/80	Male	20–55				Peer education, lecture, propaganda	②
Zhang HM	2011	Shanghai (Minxing)	400/400	female	15–49				Promotional material, video, consults	①
Hou CH	2016	Guangdong (Shenzhen)	607/616	76.9/81.2	28.7/29.8	56.3/58.4	42.2/40.7	38.1/43.3	Condoms, propaganda, game, knowledge quiz, topic discussion, meeting	①③
Zhou JB	2009	Anhui (Hefei)	943/671	93.85/94.49	35.1/38.4	26.62/17.73	66.49/80.03	82.93/87.33	Propaganda, peer education, condoms, consults	①③
Xia J	2009	Anhui (Tongling)	309/487	93.2/86.0	39.2 ± 9.3/40.7 ± 8.9	7.4/5.3	90.9/94.7	90.6/93	Health education	②④
Xu XY	2006	Zhejiang (Yoangkang)	601/558	53.7/53.4	17–51	58.9/54.3		58.5/56.6	Promotional material, knowledge contest, training, consults	③⑤
Zhang LH	2014	Hubei (Wuhan)	675/479	77.2	37.4 ± 9.8		82.8	72.4	Pamphlets, lecture	②③④⑤
Ni HN	2014	Zhejiang (Taizhou)	800/806	70.50/71.34		23.50/21.84	70.37/72.95	35.87/35.98	Promotional material	②④⑤
Zeng Y	2008	Chongqing (Yuzhong)	650/614	76/80	16–76	10/10.6	84.4/83.8	85.1/83.0	Promotional material, panels, video, consults, peer education, knowledge contest	②④⑤
Du JX	2011	Chongqing (Nanan)	606/600	85.31/85.67	20–50 of 85.81/87.84	11.39/8.67	84.32/85.50	82.84/84.33	Lecture, peer education, picture posters, condoms, advertising video	②④⑤
Wei JT	2010	Henan (Xihua, Weishi)	380/375	—	25–40 of 58.2/57.1	38.4/33.9	54.0/57.3	51.8/50.4	Lecture, pamphlets, peer education	⑤
Xiao Q	2008	Beijing	3020/3165	30/30.02	16–40 of 90	50	—	60	Peer education, brochures, panels, lectures, knowledge contest	⑤
Yang GT	2007	Shanxi (Jincheng)	200/200	100	20–60 of 86.27	6.86	93.14	74.51	Knowledge contest, educational film, condoms	①③④⑤
Liu XR	2010	Tianjin (Jixian)	345/367	100/100	20–40 of 68.4/71.66	20.29/15.53	71.01/76.84	84.35/88.01	Lecture, promotional material, posters, consults, peer education	①

Note: ①: sexual transmission of HIV; ②: condom use for HIV prevention; ③: changing attitude towards HIV/AIDS patients; ④: incidence of commercial sexual behaviors; ⑤: recent condom use during sex.

### Statistical analysis

Odds ratios (ORs) were used as the measure for summary statistics of the efficacy of the HIV intervention. Owing to the initial assumption of between-study heterogeneity, a random effects model, which incorporates both within- and between-study variability, was applied to combine the log OR across studies. A series of subgroup analyses were conducted by stratifying the original studies by baseline rate, time of intervention, peer education, region, and education background. All statistical analyses were performed using Review Manager 5.3 (Cochrane). Moreover, *P*-values less than .05 were considered statistically significant. All statistical tests were two-sided.

## Results

### Study characteristics

**Fig 1.**
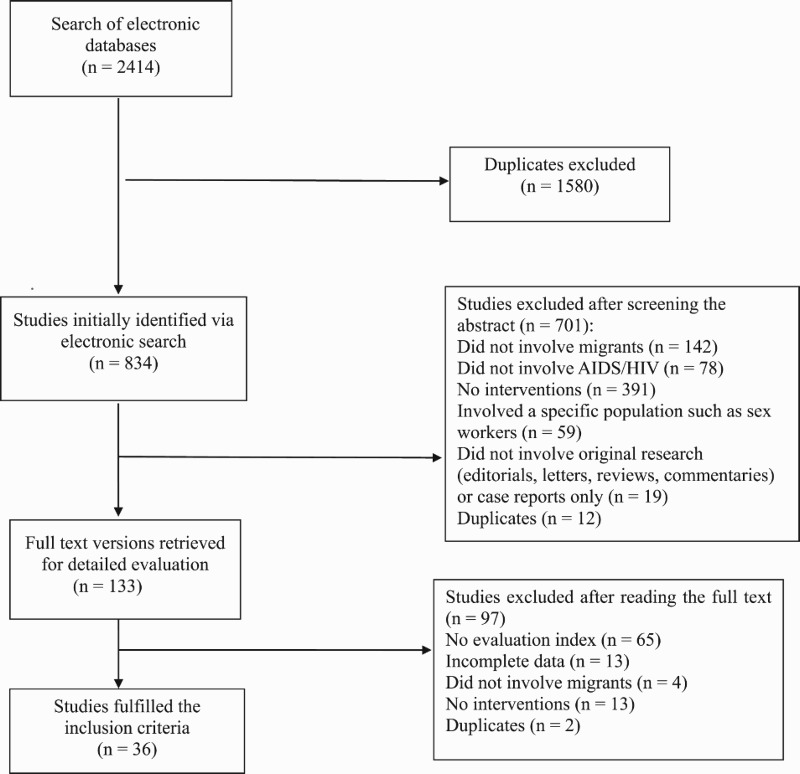
The QUOROM statement flowchart for study selection.

Our search of the electronic databases revealed 2414 studies, of which 1580 overlapped between different search categories. The search strategy for the 834 unique references is presented in Fig. 1 as the QUOROM statement flowchart in which the detailed procedure of reference identification along with information regarding exclusion criteria applied at different stages of the selection is described. Of these, only 36 (Cui & Tang, 2012; Du & Xiong, 2011; Fei & Wu, 2008; Guo, Huang, Li, & Yang, 2009; He et al., 2010; Hou & He, 2011; Hou & Shi, 2016; Hu et al., 2007; Huang & Qiu, 2015; Li et al., 2006; Liang, 2015; Liu et al., 2010; Ma, 2015; Ni, 2014; Peng et al., 2008; Peng & Zhong, 2015; Tian et al., 2010; Tian & LI, 2015; Wang; Wang, Xiao, & Lu, 2008; Cai & Hu, 2012; Wei & Zhu, 2010; Xia; Xie & Yang, 2011; Xu; Xu et al., 2006; Xu, Wang, & Song, 2006; Zhou & He, 2011; Yang & Jiao, 2007; Xiao et al., 2009; Zhang & Chen, 2011; Zeng; Zhang & Le, 2014; Zhao & Xu, 2015; Zhong, 2006; Zhou et al., 2009) articles fulfilled the predefined inclusion criteria and were selected to be included in the analysis; all the included studies were published between 2006 and 2017.

Table 1 depicts the basic information of included articles, included author, published year, region, sample size before/after intervention, gender before/after intervention, average age before/after intervention, marital status before/after intervention, education level before/after intervention, intervention measures, and outcome indicators. Table 1 indicated that studies were conducted in 22 provinces of China, and included 28,732 participants before the intervention and 24,381 participants after the intervention. The participants aged 15–65 years, and the average age of the participants was shown in Table 1, most of the participants were educated until junior high school or less. They are engaged in construction and manufacturing industries, and they always work in construction sites and small factories. For young migrants, they were more likely to work in small factories and engaged in manufacturing industries; the work in manufacturing industries was easier than that in construction industries, but the wage was lower and they only can support themselves. However, elder migrants were more likely to work in construction sites to gain much higher wages and support their family. Intervention measures included promotional material, lectures, quizzes, peer education, and condom distribution, and the time of intervention ranged from immediately to 1 year. Furthermore, 10 studies (Huang & Qiu, 2015; Li et al., 2006; Liu et al., 2010; Ma, 2015; Peng & Zhong, 2015; Wang et al., 2008; Xie & Yang, 2011; Xu, Wang, et al., 2006; Zhao & Xu, 2015; Zhou & He, 2011) did not report the duration of the intervention.

### Overall results

The overall results of this meta-analysis provided evidence in support of HIV interventions having a significant effect for all five indicators, and heterogeneity was observed for all five indicators (*P* < .01; Table 2). Seventeen studies (Fei & Wu, 2008; Guo et al., 2009; He et al., 2010; Hou & Shi, 2016; Hu et al., 2007; Li et al., 2006; Liang, 2015; Liu et al., 2010; Peng et al., 2008; Tian et al., 2010; Wang et al., 2008; Xu; Xu, Wang, et al., 2006; Zhang & Chen, 2011; Yang & Jiao, 2007; Zhong, 2006; Zhou et al., 2009) reported that the route of transmission of HIV infection was sexual, and the analysis showed that the awareness rate increased after the intervention (OR = 3.00; 95% confidence interval [CI] = 2.19–4.10; *P* < .01). Fifteen studies (Du & Xiong, 2011; Hou & He, 2011; Hu et al., 2007; Huang & Qiu, 2015; Ma, 2015; Ni, 2014; Peng & Zhong, 2015; Tian & LI, 2015; Xia; Xie & Yang, 2011; Xu, Wang, et al., 2006; Zeng; Zhang & Le, 2014; Zhao & Xu, 2015; Zhou & He, 2011) reported significantly increased awareness regarding the use of condoms for HIV prevention (OR = 4.82; 95% CI = 3.19–7.30; *P* < .01). Fifteen studies (Cui & Tang, 2012; Fei & Wu, 2008; Guo et al., 2009; Peng et al., 2008; Peng & Zhong, 2015; Tian et al., 2010; Tian & LI, 2015; Wang; Xu et al., 2006; Xu; Hou & Shi, 2016; Yang & Jiao, 2007; Zhang & Le, 2014; Zhao & Xu, 2015; Zhou et al., 2009) reported a significant change in attitude towards HIV/AIDS patients after the intervention (OR = 2.97; 95% CI = 2.23–3.94; *P* < .01). Ten studies (Du & Xiong, 2011; Ni, 2014; Tian & LI, 2015; Wang; Xia; Xie & Yang, 2011; Yang & Jiao, 2007; Zeng; Zhang & Le, 2014; Zhou et al., 2009) reported a decrease in the incidence of commercial sexual behaviors, but this decrease was not obvious (OR = 0.57; 95% CI = 0.36–0.90; *P* < .01). Lastly, 12 studies (Du & Xiong, 2011; Fitzgerald et al., 2003; Ni, 2014; Cai & Hu, 2012; Wei & Zhu, 2010; Xie & Yang, 2011; Xu et al., 2006; Yang & Jiao, 2007; Zeng; Xiao et al., 2009; Zhang & Le, 2014) reported an increase in the recent use of condoms during sex after the intervention (OR = 4.20; 95% CI = 2.43–7.26; *P* < .01).

**Table 2.** Comprehensive quantitative analysis results of the indicators.

Outcome	Number of records	*Z*-value	OR (95% CI)	*I*²
① Sexual transmission of HIV	17	6.86	3.00 (2.19–4.10)	0.92
② Condom use for HIV prevention	15	7.44	4.82 (3.19–7.30)	0.96
③ Changing attitude towards HIV/AIDS patients	15	7.51	2.97 (2.23–3.94)	0.95
④ Incidence of commercial sexual behavior	10	2.40	0.57 (0.36–0.90)	0.93
⑤ Recent condom use during sex	12	5.14	4.20 (2.43–7.26)	0.98

### Sensitivity analysis

We used a fixed effects model to combine the ORs of the five indicators, and the effect size was similar to the results of the random effects model. Thus, the combined result had good stability, indicating that the results were reliable.

### Stratified analyses

No single methodological quality feature was associated with intervention efficacy. However, the intervention effects did vary by intervention characteristics. We performed a stratified analysis according to baseline rate, time of intervention, peer education, region, and education background; the results showed that they are all factors that influence the efficacy of intervention, but the efficacy of the intervention differed according to these factors (Table 3).

**Table 3:** Subgroup-stratified analysis of indicators.

Outcome	Type	Group	*K*	Homogeneity test		Estimation of effectiveness	
				*χ*^2^	*P*	OR (95% CI)	*P*
Sexual transmission of HIV							
	Baseline rate	<60%	4	100.03	.000	2.76 (1.35–5.62)	.005
		60%–80%	6	104.54	.000	4.78 (2.16–10.55)	.000
		>80%	7	8.56	.20	2.35 (1.91–2.88)	.000
	Time (month)	<3	5	85.97	.000	2.83 (1.55–5.18)	.001
		3–6	7	116.74	.000	3.77 (1.89–7.50)	.0002
		>6	2	2.63	.1	2.40 (1.54–3.75)	.000
	Peer education	Yes	2	22.45	.000	7.42 (0.89–61.87)	.06
		No	15	165.21	.000	2.67 (1.97–3.61)	.000
	District	East	11	30.64	.0007	1.89 (1.57–2.27)	.000
		Central	5	24.65	.0001	7.68 (4.33–13.63)	.000
		West	1			5.07 (3.66–7.01)	.000
	Junior high school and below	<50%	4	19.30	.000	2.73 (1.60–4.68)	.000
		50%–80%	2	19.49	.000	5.04 (0.33–77.30)	.25
		>80%	4	80.01	.000	4.13 (1.29–13.25)	.02
Use of condoms for HIV prevention							
	Baseline rate	<60%	8	189.00	.000	6.06 (3.52–10.41)	.000
		60%–80%	3	109.86	.000	5.19 (1.54–17.46)	.008
		>80%	4	13.07	.004	2.79 (1.57–4.97)	.001
	Time (month)	<3	2	8.81	.003	4.25 (1.61–11.26)	.004
		3–6	3	48.84	.000	7.70 (3.03–19.59)	.000
		>6	3	0.67	.71	2.37 (2.05–2.74)	.000
	Peer education	Yes	4	93.44	.000	3.66 (1.46–9.15)	.005
		No	11	289.62	.000	5.29 (3.21–8.73)	.000
	District	East	6	183.26	.000	3.60 (1.53–8.47)	.003
		Central	4	33.83	.000	6.70 (3.61–12.45)	.000
		West	5	112.26	.000	5.08 (2.67–9.67)	.000
	Junior high school and below	<50%	3	12.00	.002	3.15 (1.62–6.13)	.000
		50%–80%	5	68.57	.000	8.67 (4.81–15.63)	.000
		>80%	6	146.93	.000	4.13 (2.34–7.27)	.000
Changing attitude towards HIV/AIDS patients							
	Baseline rate	<50%	8	133.89	.000	3.17 (2.24–4.49)	.000
		≥50%	7	108.86	.000	2.76 (1.64–4.64)	.000
	Time (month)	<3	4	62.04	.000	2.39 (1.42–4.02)	.001
		3–6	7	29.26	.000	2.73 (2.15–3.46)	.000
		>6	2	31.47	.000	1.97 (0.85–4.58)	.11
	Peer education	Yes	2	0.71	.40	2.35 (1.99–2.77)	.000
		No	13	254.36	.000	3.11 (2.22–4.34)	.000
	District	East	9	126.95	.000	2.30 (1.66–3.18)	.000
		Central	4	80.36	.000	4.56 (2.05–10.14)	.000
		West	2	16.05	.000	4.21 (1.57–11.28)	.004
	Junior high school and below	<50%	3	50.06	.002	3.33 (1.48–7.52)	.004
		50%–80%	8	132.79	.000	2.91 (1.91–4.43)	.000
		>80%	3	19.39	.000	3.78 (2.30–6.21)	.000
Incidence of commercial sexual behavior							
	Baseline rate	<10%	5	14.55	.006	0.95 (0.58–1.54)	.83
		≥10%	5	74.85	.000	0.39 (0.21–0.72)	.003
	Time (month)	<3	1			0.94 (0.74–1.21)	.65
		3–6	5	43.53	.000	0.28 (0.15–0.53)	.000
		>6	3	3.50	.17	1.29 (0.90–1.86)	.16
	Peer education	Yes	5	93.06	.000	0.56 (0.23–1.36)	.20
		No	5	23.70	.000	0.63 (0.39–1.01)	.05
	District	East	2	15.42	.000	0.72 (0.27–1.93)	.51
		Central	4	42.87	.000	0.24 (0.08–0.66)	.006
		West	4	8.67	.03	1.05 (0.73–1.49)	.80
	Junior high school and below	≤80%	5	31.03	.000	0.64 (0.43–0.96)	.03
		>80%	5	94.95	.000	0.47 (0.14–1.57)	.22
The recent condoms use during sex							
	Baseline rate	<20%	2	96.20	.000	15.99 (1.05–244.00)	.05
		20%–40%	5	321.68	.000	2.85 (1.14–7.08)	.02
		>40%	5	39.66	.000	3.63 (2.52–5.24)	.000
	Time (month)	<3	1			2.74 (1.82–4.14)	0.000
		3–6	5	207.17	.000	3.10 (1.55–6.20)	.001
		>6	5	319.73	.000	7.00 (2.33–21.02)	.0005
	Peer education	Yes	7	480.80	.000	4.31 (2.06–8.99)	.20
		No	5	136.73	.000	4.06 (1.81–9.10)	.0007
	District	East	5	635.76	.000	5.90 (1.92–18.14)	.002
		Central	3	5.89	.05	3.85 (2.70–5.48)	.000
		West	4	29.65	.000	2.87 (1.81–4.55)	.000
	Junior high school and below	≤80%	8	427.50	.000	3.46 (1.84–6.51)	.000
		>80%	4	219.34	.000	6.23 (1.77–21.94)	.004

## Discussion

This study investigated the efficacy of interventions from three aspects, HIV/AIDS knowledge, attitude, and behavior, using five indicators. Sexual transmission has become the major route of HIV transmission in China (Ministry of Health of the People’s Republic of China, The Joint United Nations Program on HIV/AIDS, World Health Organization, 2012); therefore, we selected HIV/AIDS transmission route and prevention method as indicators 1 and 2. Social inclusion of patients with AIDS is key for HIV/AIDS prevention, and acceptance of such migrants had a positive effect on the prevention of AIDS; thus, changing the attitude towards these patients was selected as indicator 3. The ultimate goal of the intervention was to promote people to make behavioral changes; therefore, the incidence of commercial sexual behaviors and recent condom use were selected as indicators 4 and 5, to serve as indicators of behavioral change. In addition, because of differences in survey instruments in each study, we selected the most commonly used indicators in order to improve the utilization rate of data and reduce the bias.

We were very encouraged to find that the HIV interventions were efficacious, not only in terms of improving knowledge but also in terms of attitude and behavior among migrants in China. However, after stratification by influencing factors, different factors seemed to have different effects on intervention efficacy. Our study selected articles from the past 10 years, and systematically evaluated intervention methods; we found that the efficacy of the intervention differed by baseline rate, time of intervention, peer education, region, and education background.

We found that interventions with lower baseline rates were more efficacious at improving HIV/AIDS knowledge, attitude, and behavior than interventions with higher baseline rates (Table 3). Therefore, interventions should be designed differently if the target population involves people with low awareness or those never accepted in the society; this intervention would not be very effective for people with high awareness, unless targeted measures would be used. For behavioral indicators, the longer the duration of the intervention, the better the efficacy (Table 3); we found that interventions lasting 3–6 months was more efficacious at improving HIV/AIDS knowledge and attitude than interventions lasting fewer than 3 months or more than 6 months. Thus, people would be tired of lengthy health education interventions. The optimum duration should be selected on the basis of the target population’s knowledge and attitude. An intriguing finding was that interventions conducted in developing central and western regions were more efficacious than in developed eastern regions (Table 3) for knowledge and attitude indicators. The reason for this discrepancy is that eastern cities are more liberal, AIDS education has been increasingly publicized, and the people thus have relevant knowledge. Accordingly, the effect of the intervention is not obvious. Compared to the east, the central and western regions are closed and backward, and health education is not often publicized; thus, an educational intervention can effectively improve people’s knowledge of AIDS. In future, studies should pay attention to the economic status of the area in which an intervention is planned. However, behavioral interventions in developing central and eastern regions were more efficacious than those conducted in western regions (Table 3), which may be due to the developed economy in eastern regions, where people have a higher education and more knowledge. Moreover, people in eastern regions are more likely to change their behaviors after health education, which is related to the high baseline rate in terms of HIV/AIDS knowledge and attitude. Education background also has an effect on intervention efficacy (Table 3): the interventions were more efficacious in people educated until junior high school and below in terms of the behavioral indicators, which suggested that they preferred to change their behaviors after the health education. Regarding the interventions for the knowledge and attitude indicators, the intervention effects were greater for people educated until junior high school and below, who accounted for 50%–80% of the population; thus, there was an obvious increase in HIV/AIDS knowledge and attitude among people with low education who readily accepted the health education. However, for people with extremely low education, though the intervention efficacy was greater than in people with high education, the poor cultural bias and weak background knowledge significantly negatively impacted the efforts at improving AIDS/HIV knowledge and attitude. Therefore, the intervention methods should be designed according to the target population. We need to use popular and easy ways to intervene for people with low education backgrounds, to make them easily accept AIDS/HIV-related knowledge and change their HIV/AIDS-related attitudes. In terms of peer education, there were some differences compared to that observed in previous studies. Our study findings were similar to those of Ngo, Ha, Rule, and Dang (2013), who reported that peer education is an effective means of preventing AIDS. Bond and Wolf (1998) also thought that people with a similar age, background, social economic status, and sex can have a common topic of discussion, and thus share information, which is the goal of education. Our study suggested that the efficacy of peer education as an intervention was uncertain and that the efficacy of peer education needs further study in light of the changes in HIV epidemiology.

Some achievements have been observed after intervention; however, with an increase in the number of people with AIDS, the results are different: the epidemiology of HIV had changed, along with the knowledge and attitude of migrants with HIV/AIDS. Thus, the existing intervention methods are no longer relevant; we should explore more precise measures and adopt different methods according to different people and districts, so as to design precise and optimum interventions. For example, with the widespread of social media, including WeChat, QQ, and Weibo, the internet intervention was widely used in HIV/AIDS health education. We can make a personalized intervention on HIV/AIDS among young migrants through social media. Additionally, for old migrants, more paper-made health education materials were needed, and easier, interesting paper-made propaganda was carried out for migrants with low education. For the developed eastern region, convenient network gives the chance to implement health education through social media, and intervention should include more strategies about how to reduce high-risk behavior of HIV/AIDS, while more knowledge about the harm of HIV/AIDS should be included in the health education in the developing western region.

Considering that most of the migrants had a junior high school degree, HIV/AIDS health education should be strengthened in the future, especially in rural areas and junior high school students, with an aim to enable them to recognize the threat of AIDS and learn how to protect themselves. Learning from the experiences of some countries such as Cambodia, AIDS prevention interventions should be strengthened in rural areas, including establishing farmers’ life school, paying more attention to migrant families, as well as improving AIDS-related knowledge and urban life skills training within rural communities (United Nations Development Program, 2002). These methods can make farmers acquire the necessary city life skills and health knowledge, and reduce the high-risk behavior typically observed; furthermore, this approach could effectively prevent HIV transmission within the hometowns of these migrants upon their return (Haour-Knipe, 2009).

This is the first time to evaluate the efficacy of HIV intervention in Chinese migrants. Several reviews (Crepaz et al., 2006; Crepaz et al., 2014; Globerman et al., 2017) studied the efficacy of HIV preventive interventions for people living with HIV in high-income settings, these studies focused on the specific methods of intervention among HIV patients, suggested that comprehensive risk counseling and services, provided skills building were effective in reducing sexual risk behaviors. While our study focused on the overall efficacy of HIV/AIDS interventions and the influencing factors of efficacy among migrants in China, our study suggested that the intervention conducted among migrants in China was effective in altering their knowledge, attitude, and behaviors on HIV/AIDS. Additionally, in China, the methods of intervention were numerous, including panel, video, consults, knowledge contest, lecture, etc., and no consistent criteria are there to classify these methods of intervention; therefore, it is difficult for us to evaluate the efficacy of specific methods of intervention. Maybe we can carry out a meta-analysis that aims to evaluate the efficacy of intervention methods on HIV in a further study. Futhermore, comprehensive risk counseling and services, provided skills building, group-level intervention could be carried out in the intervention of HIV/AIDS prevention in future intervention studies.

Some limitations of this review warrant comment. Our selection criteria were rather tight, which resulted in a limited number of studies. After the stratified analysis, the heterogeneity within most subgroups still existed. Prompting factors influencing the efficacy of HIV/AIDS interventions for migrants are more complicated. The composition of the migrant population is complex: different careers, flow areas, education background, workplace, age, and income are likely to impact the HIV/AIDS knowledge received, which could be the cause of the heterogeneity. Due to the mobility of this population, interventions are widespread and thus difficult to track, which leads to a large rate of missing data. This affects the evaluation of the intervention effects, especially in the long term. The majority of the studies targeted migrants in large cities; thus, it is not clear to what extent our findings are generalizable to migrants in small- and medium-sized cities.

## Conclusions

The aforementioned results corroborated the strong evidence in support of efficacy of HIV interventions for migrants in China. Further research on how interventions can be adapted to different settings and subpopulations would be valuable, especially in terms of behavior indicators.

## Ethics approval and consent to participate

The study’s protocol and data collection procedure were approved by the Institute of Public Health and Management. Our study was a meta-analysis, we collected data from published articles, we did not contact the participants, and we could not know the personal information, so our research does no harm to participants and their information was secure.

## Availability of data and materials

The datasets generated during and/or analyzed during the current study are available from the corresponding author on reasonable request.
